# On-Screen Texts in Audiovisual Input for L2 Vocabulary Learning: A Review

**DOI:** 10.3389/fpsyg.2022.904523

**Published:** 2022-05-13

**Authors:** Rong Wei, Lin Fan

**Affiliations:** ^1^School of Foreign Languages, Ma’anshan University, Ma’anshan, China; ^2^National Research Center for Foreign Language Education, Beijing Foreign Studies University, Beijing, China; ^3^School of Foreign Languages, Qingdao University of Science and Technology, Qingdao, China

**Keywords:** audiovisual input, on-screen text, subtitle, caption, L2 vocabulary, CALL

## Abstract

Audiovisual input has received increasing attention from the Second Language Acquisition (SLA) and the Computer-Assisted Language Learning (CALL) domains during the past few decades due to its vividness, authenticity, and easy accessibility. Videos with on-screen texts, as a widespread way of audiovisual input in second language (L2) teaching and learning, influence L2 learners’ performance in various aspects, including their vocabulary learning. The wide application and profound influence of such kind of input call for a systemic review on this important domain of research. Accordingly, this paper reviews the empirical studies on the effects of on-screen texts on L2 vocabulary learning. Specifically, it seeks to evaluate the role of different types of on-screen texts (i.e., subtitles, captions, and dual subtitles) and various modes of captions (i.e., full captions, keyword captions, glossed captions, annotated captions, and enhanced captions) in L2 vocabulary development. It also discusses other factors that concur with on-screen texts and influence L2 vocabulary gains from audiovisual input, such as learners’ vocabulary size, L2 proficiency, frequency of occurrence, number of viewing, instructional strategy, and test time. Finally, some suggestions are provided for future research.

## Introduction

Audiovisual input,^1^ whose application speeds up owing to the development of multimedia technology, has received growing attention from the SLA and the CALL domains during the past few decades. It not only provides vivid and authentic language learning materials, but also expands the way we access new information, even optimizes the use of our cognitive resources. Specifically, compared with traditional written input (i.e., textbooks), audiovisual input offers contextual learning environment which demonstrates language in use vividly and authentically; it is no longer limited to static information from printed words, but provides easy access to updated dynamic materials through TV, computers, and mobile devices; it conveys information that fully activates learners’ visual and auditory systems in cognition so as to optimize the learning outcome (Low and Sweller, 2014).

Audiovisual input contains several key elements, including audio, video, and on-screen text. In the fields of SLA and CALL, on-screen text is a general term for the text about the video content displayed synchronously or asynchronously on the screen (Mohsen, 2016a). It usually presents itself in different types—subtitle (L1 text, L2 video), caption (L2 text, L2 video), reversed subtitle^2^ (L2 text, L1 video) and dual subtitle (L1 + L2 texts, L2 video), or in different modes (in the case of captions)—keyword caption (L2 keywords, L2 video), glossed caption (L2 text + L1 gloss, L2 video), and so forth (e.g., Sydorenko, 2010; Lwo and Lin, 2012; Montero Perez et al., 2013, 2015, 2018; Fievez et al., 2021).

As a widespread way of audiovisual input in SLA and CALL, videos with on-screen texts have influenced L2 learners’ performance from all-round aspects, including pronunciation (e.g., Mitterer and McQueen, 2009; Wisniewska and Mora, 2020; Wong et al., 2020; Mohsen and Mahdi, 2021), grammar (e.g., Lee and Révész, 2018, 2020; Pattemore and Muñoz, 2020), writing (e.g., Alobaid, 2021), pragmatics (e.g., Barón and Celaya, 2022), listening comprehension (e.g., Montero Perez et al., 2014a; Liao et al., 2020; Pujadas and Muñoz, 2020; Lee et al., 2021), as well as vocabulary learning^3^ (e.g., Montero Perez et al., 2014b; Peters et al., 2016; Teng, 2020; Muñoz et al., 2021). The general positive role of on-screen texts was reported in these aspects. In particular, empirical research on the role of on-screen texts in L2 vocabulary learning have yielded fruitful but inconclusive results when they compared the effects of on-screen texts in various forms, which is the focus of the present review. Generally, some researchers have explored the effects of the two major types of on-screen texts and observed that captions exerted greater influence than subtitles in promoting word learning (e.g., Frumuselu et al., 2015; Peters et al., 2016; Peters, 2019; Baranowska, 2020), while others claimed that types of on-screen texts had no significant correlations with learners’ performance on vocabulary growth (e.g., Lwo and Lin, 2012; Muñoz et al., 2021). Still another group concentrating on the diversified caption modes (e.g., keyword caption, glossed caption, annotated caption, and enhanced caption) obtained even more multifarious results.

Given that researchers have yielded fruitful results of the role of on-screen texts in L2 learning, reviews were conducted to depict the research status and development in this field. Vanderplank (2010), one of the pioneers, gave an assessment of primary research studies on language teaching and learning *via* television and video and highlighted the role of subtitles and captions. Thereafter, reviews on L2 learning aided by videos with on-screen texts have emerged intermittently. Some of them were comprehensive ones which discussed multiple aspects of L2 learning (e.g., Matielo et al., 2015), while others focused on one or two specific aspects, among which listening comprehension (e.g., Montero Perez et al., 2013; Mohsen, 2016a; Yeldham, 2018) and vocabulary learning (e.g., Montero Perez et al., 2013; Mohsen, 2016a) were the most popular topics in these reviews. Nonetheless, these reviews barely offer an in-depth description about the field of on-screen-text-aided L2 learning mainly in consequence of the inadequate empirical research. Fortunately, this field has met its heyday thanks to the ever-developing multimedia technology. Empirical studies have sprouted in the past few years, which call for latest reviews on literature. Most recently, Montero Perez (2022) presents a panorama of the status quo by examining a variety of documents ranging from books and edited volumes to reviews, conferences, special issues, as well as empirical studies, and encompassing such inclusive L2 learning aspects as comprehension, vocabulary, grammar and listening. The existing reviews have undoubtedly made great contributions to the academic landscape of CALL or SLA supported by videos with on-screen texts. Since vocabulary plays a predominant role in L2 learning, as indicated in a much-cited statement that “Without grammar very little can be conveyed; but without vocabulary nothing can be conveyed” (Wilkins, 1972, p. 111), “most [empirical] studies have looked into the potential of on-screen text for vocabulary learning” (Montero Perez, 2022, p. 20). Yet there is few review specifically taking vocabulary learning as the sole object to delve into the effects of on-screen texts. To our knowledge, Teng (2021) was the only one who paid exclusive attention to incidental vocabulary acquisition from captioned videos, but only focused on 6 studies in his review.

Hence, it is urgent to launch a review to sort out the literature on the effects of on-screen texts on L2 vocabulary learning. To identify the scope of this review, several criteria were used to select the literature. First, research articles, monographs, and book chapters that employ experimental designs are included. Doctoral dissertations, conference proceedings, editorial materials, book reviews are all excluded. Second, since the application of audiovisual input to L2 learning has been greatly accelerated *via* the flourishing Internet-related platforms during the past decade, that is, from 2012 to 2022, this review mainly concentrates on but is not confined to this time span. Prominent earlier studies would also be mentioned to demonstrate their contributions to this area. Third, studies published in languages other than English are ignored. Fourth, the research area is restricted to SLA and CALL. Articles discussing the role of on-screen texts in supporting pre-school children or the deaf or hard-of-hearing, or studies addressing other issues (e.g., reading behavior or working memory) with on-screen texts merely as the context are all expunged. Last but not least, videos with on-screen texts in this review are defined as a kind of audiovisual input which is composed of dynamic visuals instead of static pictures or graphics, audio with L2 soundtrack, and different forms of on-screen texts.

Following the criteria of data retrieval, this review sets out to paint a comprehensive picture about the effects of videos with on-screen texts on L2 vocabulary learning. It begins with the theories which lay a solid foundation for research on audiovisual input in L2 learning. Then, based on the clarification of terminology and categories of on-screen texts in SLA and CALL contexts, the retrieved empirical studies are reviewed mainly in two hierarchical groups. Meanwhile, some learner-related and experiment-related factors that concur with on-screen texts to influence L2 vocabulary development are also appraised. Finally, suggestions for future research on on-screen-text-aided L2 vocabulary learning are provided.

## Rationale for Audiovisual Input in L2 Learning

### Dual Coding Theory

The origin of dual coding theory (DCT) can be traced back to Paivio (1969) review on the studies of imagery. Paivio (1990) believed that humans are unique in nature in that they have the innate ability to deal concurrently with modality-specific verbal and non-verbal representations which herein refer to language and imagery, respectively. Accordingly, the fundamental assumption in DCT is that human cognition in reading and writing depends on dual coding systems of mental representations. The one in charge of the verbal representations is defined as the verbal system specialized for language; the other in charge of the non-verbal representations is the imagery system specialized for non-verbal objects and events (Paivio, 1990, 2010; Sadoski and Paivio, 2000). The two systems function independently but interconnectedly, that is, they can be activated individually or simultaneously, depending on the nature of the task. Both systems contribute to human cognition in reading and writing, the operating units of which are, respectively, called logogen and imagen (Paivio, 2010).

The idea of dual coding systems in handling environmental information, then, enlightened research on on-screen-text-aided L2 learning from audiovisual input. The visual information in videos forms imagens and the soundtrack along with the on-screen texts constitutes corresponding logogens, which together contribute to the process of new information in cognition. Since human cognition functions in two systems according to this theory, the combination of visual and auditory stimuli should outperform any single stimulus, thus laying a foundation for the merits of audiovisual input.

### Cognitive Load Theory

Cognitive load theory (CLT) was put forward by Sweller in 1988 based on human cognitive architecture. According to CLT, cognitive architecture is composed of “working memory, long-term memory and the relationships between them” (Sweller et al., 2019, p. 263). The process of learning is largely confined to our working memory which can only handle a limited number of information elements for a limited duration (*ibid.*). Hence, the more requirements a learning task imposes, the higher the cognitive load is. When it is beyond the capacity of the working memory, it hinders information transformation and knowledge construction into the long-term memory (*ibid.*). On the other hand, if the cognitive resources are reasonably allocated to information from different modalities (i.e., visual and auditory), cognitive load will be reduced and learning will be more effective than from a single modality (Low and Sweller, 2014). As a result, the modality effect, one of the cognitive load effects, also lends support to audiovisual input for L2 learning.

### Multimedia Learning Theory

Multimedia learning theory proposed by Mayer is closely associated with the above two theories. It hypothesizes that “people can learn more deeply from words and pictures than from words alone” (Mayer, 2014, p. 1). This theory entails three assumptions: the dual-channel assumption (i.e., there are two channels for processing visual and auditory information separately), the limited capacity assumption (i.e., each channel has a limited capacity), and the active processing assumption (i.e., humans are active processors of the ongoing information; *ibid.*). It provides an explanation to the cognitive processes of language learning from audiovisual input: learners first select useful words and images, then organize them into coherent verbal and pictorial representations, and finally integrate these representations with their existing knowledge (*ibid.*). In this theory, multimedia instruction principles inspired by cognitive load effects (e.g., the split-attention effect, the redundancy effect, and the modality effect) were elaborated and thereafter have shown far-reaching significance in explaining the various outcomes of empirical research on L2 learning from audiovisual input.

## Categories of On-Screen Texts

Even though audiovisual input has been applied to L2 teaching and learning for a few decades, the divergence in using terminology has not been settled yet. Some studies did not make a distinction between “subtitle” and “caption.” Instead, they commonly employed “L1/L2 subtitles” (e.g., Bisson et al., 2014; Vulchanova et al., 2015; Baranowska, 2020; Hao et al., 2021), or occasionally “L1/L2 captions” (e.g., Lwo and Lin, 2012) to refer to interlingual and intralingual on-screen texts, respectively. However, this phenomenon may lead to confusion for readers and novice researchers.

To clarify these two concepts, we need to summarize the history of their applications first. Initially, subtitle was one of the favorite adaptation methods in European countries in the 1990s when people were frequently introduced to foreign language TV programs (Koolstra et al., 2002). It presented (condensed) translation in the viewers’ native language along with the original foreign language soundtrack (Peters et al., 2016). Gradually, its value in fostering informal language learning was detected (*ibid.*). By contrast, caption was, originally, not designed to oblige the common people, but rather the deaf or hard-of-hearing (Vanderplank, 2010; Bisson et al., 2014; Peters et al., 2016; Teng, 2021). Like subtitle, caption was then regarded as a scaffold to facilitate L2 learning provided that the input was not too challenging for the learners’ language proficiency (Danan, 2004; Vanderplank, 2010). While the effect of subtitles on L2 learning was widely scrutinized in earlier research carried out in the 1990s, caption has been under the spotlight as a more recent concern (Peters et al., 2016). Consequently, various terms, derived from different caption modes, were engendered to meet their corresponding research purposes.

**Figure 1:**
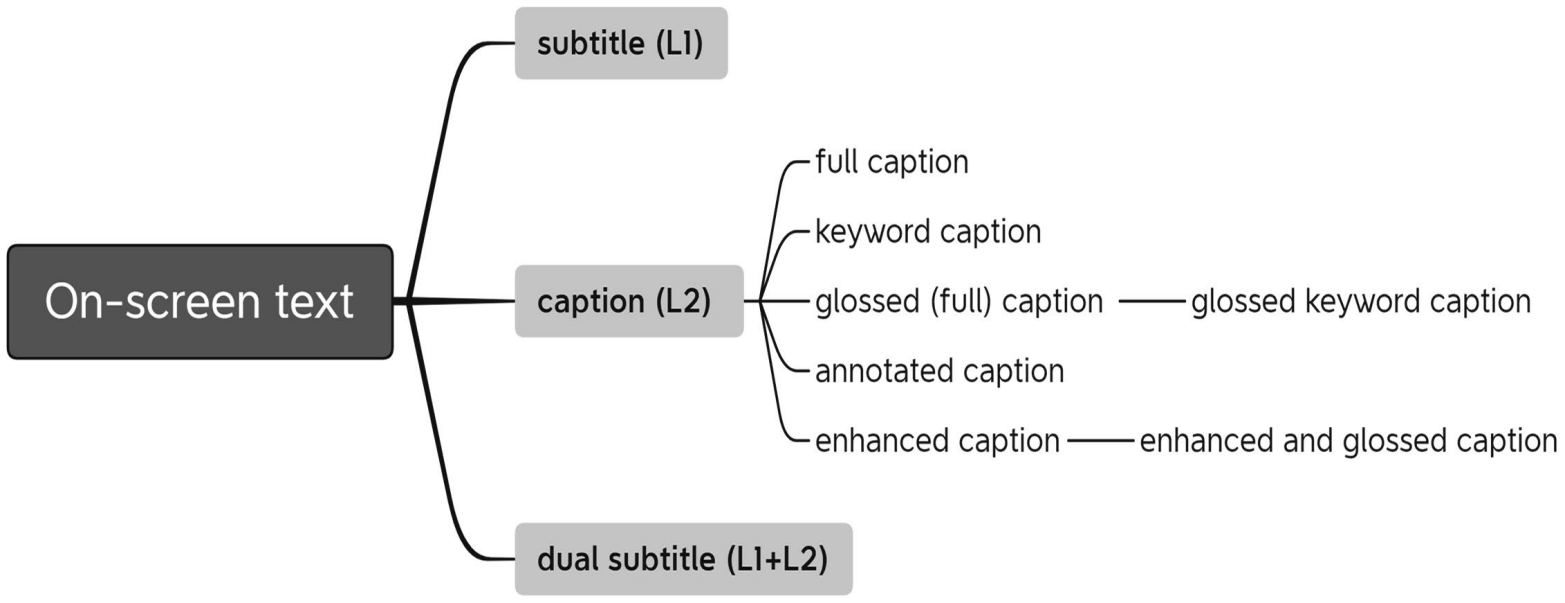
Categories of on-screen texts and their relations.

This review tentatively categorizes the empirical studies into two groups according to the hierarchical order of their involved on-screen texts. The one on the upper level pertaining to different types of on-screen texts (i.e., subtitle, caption, and dual subtitle) concerns the optimum language(s) to be displayed with videos; and the other on the lower level of different modes of captions (i.e., full caption, keyword caption, glossed caption, annotated caption, and enhanced caption) is to further investigate the favorable mode of displaying one particular type of on-screen text—caption, which is generally supposed to be beneficial. In SLA and CALL contexts, we adopt the definitions prevailing in most studies. Subtitle is the interlingual on-screen text which provides L1 translation to the L2 soundtrack, and caption is the intralingual on-screen text which provides L2 verbatim transcription to the L2 soundtrack (Danan, 2004; Winke et al., 2010; Hsu et al., 2013). Dual subtitle is the one that combines L1 translation and L2 verbatim transcription simultaneously (Lwo and Lin, 2012; Hao et al., 2021; Wang and Pellicer-Sánchez, 2022). Full caption, another term for caption, is employed when it is discussed in the scope of caption modes to differ from keyword caption in particular. Keyword caption is the caption mode which only contains one or a maximum of three or four consecutive words that are crucial for constructing sentence meaning (Montero Perez et al., 2018; Teng, 2019). Glossed (full) caption is defined as complete captions in which some keywords include access to contextual meaning in viewers’ native language (Teng, 2020). Annotated caption is a kind of full caption with some keywords connected to an annotation which contains the L1 and/or L2 definition(s) of the word, an L2 example sentence, an image for illustration, and sometimes the words’ pronunciation (Aldera and Mohsen, 2013; Mohsen, 2016b). Enhanced caption is a kind of full caption with some keywords bolded, underlined, or colored to enhance their salience (Cintrón-Valentín and Garciá-Amaya, 2021; Majuddin et al., 2021). Figure 1 shows the main categories of on-screen texts and their relations.

## Effects of On-Screen Texts on L2 Vocabulary Learning

It is acknowledged that on-screen texts do exert a positive influence on L2 vocabulary learning. Yet researchers have never stopped moving forward to explore the most beneficial language to be displayed and the most appropriate way of displaying the language on the screen. In line with the foci of the empirical studies, we attempt to answer the above two questions by investigating, respectively, the effects of different types of on-screen texts and the effects of different modes of captions. Since word knowledge is a multi-dimensional concept that involves form, meaning, use, and each of them can be further divided into more detailed branches (for details, see, e.g., Teng, 2021), the retrieved empirical studies may explore one or several aspects of word knowledge so as to contribute to the whole view of on-screen-text-aided L2 vocabulary learning.

### Effects of Different Types of On-Screen Texts

The positive effects of subtitles (e.g., Koolstra and Beentjes, 1999) and captions (e.g., Neuman and Koskinen, 1992; Yuksel and Tanriverdi, 2009; Sydorenko, 2010; Winke et al., 2010) on vocabulary growth were identified separately in earlier studies. Recently, researchers tend to figure out which type of on-screen text (i.e., language to be displayed on the screen) is the optimum for L2 vocabulary gains. In addressing this issue, three camps were formed according to their research results (Table 1).

**Table 1:** Three camps in addressing the optimum type of on-screen text.

	Synchronic studies	Longitudinal studies
Camp 1 (Captions were better)	Peters et al., 2016	Frumuselu et al., 2015
	Peters, 2019	
	Baranowska, 2020	
	Wang and Pellicer-Sánchez, 2022	
Camp 2 (Irrelevant to types of on-screen texts)	Lwo and Lin, 2012	Frumeselu, 2019
	Bisson et al., 2014	Muñoz et al., 2021
	Vulchanova et al., 2015	
	Birulés-Muntané and Soto-Faraco, 2016	
Camp 3 (Subtitles were better)		Hao et al., 2021

The first camp held that captions were superior to subtitles. Peters et al. (2016) carried out two experiments, respectively, on intermediate and low-proficiency English-as-a-foreign language (EFL) students to investigate the differential effects of subtitles and captions. The two experiments almost arrived at the same conclusion that captions showed greater influence on word form than subtitles. However, the results may be partially explained by the fact that participants’ unfamiliarity with captions aroused their additional attention to the form of the displayed target words. Despite the small sample size and the general low learning gains in all groups, this study provided enlightening directions for future research, such as the combination of captions and imagery, which illuminated a subsequent experiment of great importance in this field (i.e., Peters, 2019). Peters (2019) and Baranowska (2020) which invited intermediate EFL students as their participants both showed that the caption groups performed better than the subtitle groups and the control groups without any on-screen text. A more recent study conducted by Wang and Pellicer-Sánchez (2022) supplemented a less discussed type—dual subtitle—while comparing the effects of different on-screen text types. They found that captions significantly outperformed the other three types (i.e., dual subtitle, subtitle, and no subtitle) in form recognition, and dual subtitles showed greater potential in promoting meaning recall and meaning recognition. This eye-tracking study not only demonstrated the positive role of dual subtitles on meaning knowledge, but also confirmed participants’ preference for L1 translation to L2 form in dual subtitles with online evidence. A longitudinal study (i.e., Frumuselu et al., 2015) tested the informal and conversational speech learning outcome of university undergraduates with different L2 proficiency from A2-C1 of CEFR after a 7-week intervention. The results again confirmed the advantage of captions, independent on L2 proficiency.

The findings of the second group showed that L2 vocabulary learning had little to do with types of on-screen texts. Lwo and Lin (2012) were among the few researchers who introduced dual subtitles into their study, yet their result was totally different from that in Wang and Pellicer-Sánchez (2022). Instead of observing the positive impact of captions or dual subtitles, they found that neither the existence nor the types of on-screen texts exerted any influence on junior high school students’ vocabulary recognition and use, which was ascribed to the excessive visual and auditory support that dwarfed the effects of on-screen texts in the teaching material. In other words, the audiovisual input was so easy that the learners did not rely on the on-screen texts anymore. Contrary to the easy input, more studies showed no vocabulary gains from audiovisual input irrespective of the on-screen text types under the circumstances that the participants were completely new to the target L2 (Bisson et al., 2014) and that the input full of low frequency words was far beyond the learners’ linguistic ability (Birulés-Muntané and Soto-Faraco, 2016). These may indicate that the on-screen texts would turn out to be in vain when the input is either too easy or too difficult. So the above synchronic results may be untenable as a consequence of the inappropriateness of learning materials. Vulchanova et al. (2015) compared the long-term effect of subtitles and captions by measuring learners’ word definition and word recall performance 4 weeks after viewing the learning material. Although an age-modulated effect was noticed in the subtitle condition in word definition task, no effects of on-screen text types were observed in separate age group analyses in either word definition or word recall. The discouraging result was ascribed to two reasons: the long interval between the viewing and the tests, and the one–off intervention which provided no more encounters. In addition, it was worth mentioning that the research of Frumeselu (2019), almost identical to that of Frumuselu et al. (2015) in experimental design, reported that neither subtitles nor captions stimulated the learning of informal and colloquial vocabulary, which seemed to violate the earlier conclusion. Practically, this contradiction was attributed to the only difference in testing procedure, that is, the immediate tests administered after each viewing session in the later study did not allow students much time to internalize the knowledge. This explanation implied that “gaining benefits in terms of language acquisition appeared to be a lengthy process” (Vanderplank, 2016, p. 238–239) and, meanwhile, emphasized the importance of long-term intervention. The invalidity of on-screen texts on vocabulary learning was subsequently confirmed as a result of the low L2 proficiency in a year-long study (i.e., Muñoz et al., 2021).

Hao et al. (2021) were the representatives in the marginal third camp who believed that subtitles were more beneficial than captions in L2 vocabulary learning. They conducted another experiment that brought in dual subtitles and found that subtitles outperformed captions among the advanced learners after a 2-week intervention. However, it should be noticed that the control group without any on-screen text was the best of all, followed by the dual subtitle, the subtitle, and the caption groups in descending order, which denied the effects of on-screen texts on the whole among these advanced learners. This exceptional outcome was then ascribed to the redundancy effect (see Hao et al., 2021).

Despite the controversies in the experimental results, a tendency can still be spotted: more researchers tend to believe that subtitles show no advantage over captions in L2 vocabulary learning. A widely accepted explanation is that captions can help learners segment the speech stream in the L2 soundtrack and facilitate form-meaning mapping (Winke et al., 2010; Peters et al., 2016; Peters, 2019; Hsieh, 2020; Montero Perez, 2022), while subtitles cannot. As regards the second camp, researchers may attribute the inefficiency to learners’ low proficiency or different testing procedure. However, the participants’ L2 proficiency in these experiments was from beginning to intermediate. While some scholars may hold that subtitles are more suitable for beginners and captions for higher-level learners (e.g., Vanderplank, 2010), it is noted that appropriateness is the basic law in selecting learning materials and testing procedures. “Concerns about whether lower-level students can benefit from captions in the same way as upper-level learners may be more about the appropriateness of the video’s complexity level for the lower-level learners rather than the appropriateness of the captioning for lower-level learners” (Winke et al., 2010, p. 80). Therefore, the difficulty of audiovisual input, as a relative concept, should be, alongside the testing procedure, adapted to match or be slightly above the learners’ ability. If so, a different picture may be unfolded. To date, the role of different types of on-screen texts is yet to be further explored, especially the newly emerging dual subtitle, whether it may turn out to be the optimum type of on-screen text which incorporates the advantages of both subtitles and captions, or a redundant type which distracts learners’ limited cognitive resources.

### Effects of Different Modes of Captions

Apart from the optimum language to be displayed on the screen, the way of displaying the text is another issue of concern. Given the abovementioned advantages of captions, there has been an inclination toward an in-depth investigation into the effective way of displaying this type of on-screen text among scholars. They put forward two main approaches to make captions more accessible so as to maximize their functions: one was to reduce the captions on-screen, which resulted in a concise mode, namely, keyword caption; the other was to enrich the captions, which gave rise to the modes of glossed (full) caption, annotated caption, and enhanced caption. These modes, rising in response to their corresponding research purposes, may integrate one another in experiments and generate more variations, such as glossed keyword caption (i.e., keyword caption with corresponding L1 context-bound translation to each individual word; e.g., Hsu et al., 2013; Montero Perez et al., 2018; Teng, 2020) or enhanced and glossed caption (i.e., full caption with heighted target words and their contextual L1 translation; e.g., Hsieh, 2020). Due to the diversification of caption modes, we classify the empirical studies based on their methodology (i.e., caption modes) instead of the research results (Table 2).

**Table 2:** Classification of studies based on caption modes.

Caption modes	Empirical studies	Effects of caption modes (in declining order)
First direction (reduction)		
Keyword captions (KC)	Montero Perez et al., 2015	KC, FC
	Teng, 2019	FC, KC, 0
Second direction (addition)		
Glossed (full) captions (GFC)/ glossed keyword caption (GKC)	Montero Perez et al., 2018	GKC, KC, FC, 0
	Teng, 2020	GFC, FC, GKC, KC
	Hsu et al., 2013	GFC, GKC, 0
Annotated captions (AC)	Aldera and Mohsen, 2013	AC, FC, 0
	Mohsen, 2016b	AC, AT
Enhanced captions (EC)	Montero Perez et al., 2014b	EC, KC, FC, 0
	Hsieh, 2020	EGC, FC, EC, 0, FCNA
	Cintrón-Valentín and Garciá-Amaya, 2021	EC, 0
	Majuddin et al., 2021	EC, FC, 0

*FC, Full caption; 0, No caption; AT, Annotated transcript; EGC, Enhanced and glossed caption; and FCNA, Full caption without audio.*

The efficiency of the first approach to reduce captions was a major concern among scholars. As a result, the comparison between full captions and keyword captions has been the fundamental part, to a certain extent, in the bulk of captioning studies. Montero Perez et al. (2015) compared the effects of these two major caption modes and evaluated the (high-) intermediate proficiency learners’ vocabulary increment in a comprehensive way. They found keyword captions were more robust in promoting form recognition than full captions, but helpless in terms of clip association and meaning recall. The results indicated that visual salience *via* reduction overtly directed learners’ attention to the L2 word form, but failed to induce further form-meaning mapping which was regarded as one of the main benefits of full captions in L2 vocabulary learning. Treading on their heels, Teng (2019) supplemented a control group with no captions among a large number of primary school students. Overwhelming merits of full captions compared to keyword captions and no captions were observed in new words learning from all-around aspects, including form recognition, meaning recall, and meaning recognition (Teng, 2019). Though the studies yielded inverse results, they all verified the effect of full captions on constructing form-meaning connection in learners’ mental lexicon.

To further enhance the accessibility of captions, Montero Perez et al. (2018) and Teng (2020) extended their research to the second approach by introducing glosses to their respective previous experimental design. Montero Perez et al. (2018) added a no caption group and a glossed keyword caption group to assess learners’ vocabulary uptake. The results revealed that the glossed keyword caption group picked up most in form recognition, clip association, and meaning recall, the keyword caption and the full caption groups did not show much difference in these tests, and the no caption group was the poorest. Besides, the keyword caption group was only slightly worse than the glossed one in form recognition. The findings not only attached great importance to glosses, but also corroborated their earlier emphasis on visual salience *via* reduction (i.e., Montero Perez et al., 2015). Teng (2020), on the other hand, added one more new mode, glossed full caption, to fully tap into the competition between the full caption groups and the keyword caption groups. In line with his previous results (i.e., Teng, 2019), the full ones outperformed the keyword ones, with the scores of word form, meaning, and use ranging in a declining order: glossed full caption, full caption, glossed keyword caption, and keyword caption. It can be drawn from this study that when compared to the integrity of captions, visual salience was put in the shade in fostering vocabulary uptake, consistently contradictory to the results of Montero Perez et al. (2015, 2018). Nonetheless, the contradiction stemmed from synchronic studies. Hsu et al. (2013) observed that elementary school students in the glossed full caption and the glossed keyword caption groups shared a similar incremental pattern in their vocabulary capability in a period of 1 month, which smoothed the dispute by taking intervention duration into account.

Another line of scholars endeavored to make captions more comprehensible and conspicuous through annotations and enhancement. Aldera and Mohsen (2013) employed annotations in captioned animation to foster word learning. They assigned the high beginners to three interventional conditions: full captions, annotated captions, no captions. Annotations in this study demonstrated the utmost potential in fueling L2 word recognition and production over the short and the long term (i.e., 4 weeks). Afterward, Mohsen (2016b) compared the effects of captions and transcripts, along with annotations. The positive results of both groups confirmed the combined effects of annotations and captions/transcripts on the one hand, and the better performance of the caption group provided additional evidence to consolidate the role of captions in L2 vocabulary development on the other hand.

Montero Perez et al. (2014b) introduced enhanced captions into their research to find the best facilitating mode. They reported that full captions, keyword captions and enhanced captions all improved word knowledge in form recognition and clip association significantly compared to the control group with no captions, thus stressed the importance of captions. Meanwhile, the results also exhibited greater influence of keyword captions and enhanced captions than full captions on meaning recognition, which aligned itself with the later findings (i.e., Montero Perez et al., 2015, 2018) that salience was superior to integrity in presenting captions. However, a more recent study (i.e., Majuddin et al., 2021), which tried to minimize the negative impact of a long lapse (4 weeks) between exposure and testing, as indicated in Vulchanova et al. (2015), by shortening the interval to 2 weeks, yielded partially different results. In their study, the college students in the full caption group and the enhanced caption group with bolded or underlined target items both outscored the control group with no captions in the form recall test immediately after the viewing. However, no advantage of salience over integrity was observed since the enhanced one did not differ much from the full one. Furthermore, the positive impact of enhancement disappeared among participants with high pretest scores in the delayed test, implying a general decline of effects of enhanced captions over a certain period. And this was also the case in Cintrón-Valentín and Garciá-Amaya (2021) which demonstrated an obvious positive impact of enhanced captions in word recognition and production in the immediate posttest, but declined sharply in 2 weeks. Additionally, there was an even more mixed mode—enhanced and glossed caption—in audiovisual input. Hsieh (2020) conducted an experiment on low–intermediate EFL learners assigned to five groups, including no captions, full captions, full captions without audio, enhanced captions, enhanced and glossed captions, to examine the effects of each mode on L2 vocabulary improvement. The author found that the enhanced and glossed caption group surpassed the other groups in that it was not only prominent in form recognition, but also outstanding in meaning recognition and meaning recall. The one in the second place was the full caption group who did fairly well in the three tests, whereas the enhanced caption group only scored significantly higher than the other two groups in form recognition. These results indicated that enhancement and glosses could corporately contribute to learners’ high involvement and form-meaning link construction and that captions could be taken as a scaffold only when synchronous audio input was available.

In sum, these experiments were conducted on the assumption that captions were beneficial to L2 vocabulary learning, and all lived up to the expectations. They provide research-based evidence to support the two approaches of releasing the potential of captions to the maximum. Specifically, though there is a debate on the effectiveness of reduction (i.e., keyword caption) compared to integrity (i.e., full caption), the former always demonstrates positive impact on vocabulary learning gains. As for the accessibility of captions promoted by addition, glosses and annotations are proved to be facilitative, and the effectiveness of enhancement is obvious immediately after the viewing but declines as time goes by.

### Other Influencing Factors Concurring With On-Screen Texts

In reviewing the empirical literature, we noticed that some other factors did exert enormous influences on L2 vocabulary learning from audiovisual input, albeit the general positive impact of on-screen texts. These factors mainly include such learner-related ones as vocabulary size, L2 proficiency, and learning style, as well as experiment-related ones as frequency of occurrence, number of viewing, instructional strategy, and test time. They were sometimes specifically examined, and sometimes discussed as by-products in their experimental results. Additionally, due to the diversity of research in this area, more factors concerning learner, input, and test are only occasionally explored or even underexplored. Table 3 lists these influencing factors that possibly concur with on-screen texts.

**Table 3:** Explored, occasionally explored, and underexplored influencing factors.

	Learner-related factors	Experiment-related factors	
		Input-related ones	Test-related ones
Explored	Vocabulary size	Frequency of occurrence	Test time
	L2 proficiency	Number of viewing	
		Instructional strategy	
Occasionally explored	Learning style	Language distance	Test modality
		Captioning order	Retention interval
		Content familiarity	
Underexplored	Working memory	Duration of the viewing	Aspect of word knowledge
		Number of sessions	Test instrument
		Types of video	Task type

Among the learner-related factors, learners’ prior vocabulary knowledge and L2 proficiency are deemed to be the key elements in deciding L2 vocabulary gains from written input (e.g., Alavi and Kaivanpanah, 2009; Lee et al., 2020). Inspired by the enlightening results in previous reading studies, scholars attempted to figure it out whether it was also the case from audiovisual input. The literature in this viewing area^4^ has almost reached consensus on the positive role of vocabulary size: the more words a learner knows, the more gains the learner will harvest (e.g., Fievez et al., 2021). Vocabulary size was reported to greatly improve form recognition, clip association, and form recall and have even greater effect sizes than caption modes (Montero Perez et al., 2014b) and on-screen text types (Peters et al., 2016) for meaning recognition and recall. Additionally, some studies though only made a passing mention on vocabulary size, taking it as a by-product, all agreed upon its positive role (e.g., Montero Perez et al., 2015, 2018; Majuddin et al., 2021). The situation in L2 proficiency was a bit more complex. While proficiency was found superior to on-screen text types in predicting learners’ success in both word form and word meaning uptake (Muñoz et al., 2021), there was another view that vocabulary gains bore little relation to learners’ prior L2 proficiency (Frumuselu et al., 2015). Compared to the above two main factors, learning style is a marginal one in discussion. It consists of four dimensions, among which the visual/verbal learning style dimension is most suitable for audiovisual input. We are aware of only one study (i.e., Hsu et al., 2013) that tentatively took this dimension into consideration but did not discuss it in detail.

As to experiment-related factors, frequency of occurrence is one of the most popular topics inserted in the discussion of on-screen texts. Peters et al. (2016) observed an almost synchronous increase between the odds of learning a word and its frequency of occurrence, although frequency was dependent on vocabulary size. Besides, a number of studies which definitely examined the influence of frequency of occurrence confirmed its positive relation with gains in word form and meaning (e.g., Teng, 2019; Fievez et al., 2021; Muñoz et al., 2021). A similar factor which also underlines the role of repetition in vocabulary learning is the number of viewing, which was specifically examined in a study of multiword expressions learning initiated by Majuddin et al. (2021). The results, undoubtedly, showed the beneficial role of more encounters. In addition to the factors related to learning materials, two instructional strategies were employed to promote the learning outcome. One was test announcement strategy that announced an upcoming vocabulary test before the viewing so as to arouse learners’ attention to the unknown words in captions, which, in turn, might enhance vocabulary gains (Montero Perez et al., 2015, 2018); and the other was advance-organizer strategy, commonly used in in-class instruction, that helped learners form a conceptual framework before viewing by providing relevant background information as well as activities (Teng, 2020). While the former strategy then turned out to be less facilitative (Montero Perez et al., 2015, 2018), the latter demonstrated great potential in helping vocabulary learning (Teng, 2020). Another factor that cannot be neglected in predicting the learning outcome is something concerning test time. Practically, this factor can be subdivided into two minor ones: the interval between the intervention and the posttest (i.e., immediate or delayed) and testing procedure of the posttest (i.e., at the end of the whole study or after each session) specifically in longitudinal studies. Some studies administered both immediate posttests and delayed tests in order to reveal the durability of the observed gains (e.g., Aldera and Mohsen, 2013; Mohsen, 2016b; Cintrón-Valentín and Garciá-Amaya, 2021; Majuddin et al., 2021). Generally, the scores in these delayed tests were confronted with a sharp decline compared to the immediate posttests, but still higher than those without any intervention, which indicated that the vocabulary knowledge facilitated by the on-screen texts needs to be consolidated after viewing, probably through more regular encounters, to sustain its retention. Others paid attention to the long-term effect by administering delayed tests only (e.g., Yuksel and Tanriverdi, 2009; Vulchanova et al., 2015). Though the between-group differences were not significant, the on-screen texts still showed positive impact on word learning, which provided evidence for their supportive role in L2 vocabulary retention. Empirical results also varied with the testing procedure. The typical examples were the different results derived from two 7-week longitudinal studies which were identical in every aspect except for the testing procedure, one with a tests-at-the-end format (i.e., Frumuselu et al., 2015) and the other with a tests-after-each-session format (i.e., Frumeselu, 2019), as mentioned above (see *Effects of Different Types of On-screen Texts*). The two contradictory results revealed that vocabulary learning was a process of accumulation in which learners needed time to internalize the new information they received. More longitudinal studies (e.g., Hsu et al., 2013; Muñoz et al., 2021) tended to choose the regular testing pattern after each session which could not only record the whole learning process regularly but also reflect the learning outcome more comprehensively without missing any important turning point, especially in a long-lasting experiment.

We may find that not all the factors mentioned were definitely examined in such viewing studies, and some of them were only mentioned as by-products in their discussions. Some other factors, such as test modality, language distance between the native and the target language, captioning order, content familiarity, and retention interval, were only occasionally discussed in the context of audiovisual input (e.g., Sydorenko, 2010; Winke et al., 2010, 2013; Fievez et al., 2021). And there are still a number of factors underexplored, including such input-related ones as duration of the viewing, number of sessions, types of the video (e.g., cartoons, TV series, or documentaries), and some test-related ones like aspect of word knowledge (e.g., form/meaning/use, productive/receptive, or as a whole), test instrument (e.g., Vocabulary Knowledge Scales or Vocabulary Levels Test), task type (e.g., multiple choice, cloze, or question and answer), and so forth. Besides, there is a notable lack of viewing research on learner-related factors, for example, learning style and working memory. Each of the above factors may contribute to the growth of L2 vocabulary from videos with on-screen texts, the extent to which needs to be identified through more well-controlled examinations.

## Conclusion and Future Directions

Audiovisual input has greatly contributed to the development of CALL and the transformation of SLA from a book-based to a video-based activity. This review sets out to depict a comprehensive picture of the major findings from two lines of research in the past decade, with types of on-screen texts and modes of captions as their foci, to unveil the effects of videos with on-screen texts on L2 vocabulary learning. The major theories prevailing in CALL or SLA research under the audiovisual condition are introduced to improve the understanding of the designs and results of the studies. By scrutinizing the most relevant empirical studies, this review generalizes three major findings to reflect the status quo. First, as for the helpful type (or language) of the on-screen text, captions (L2) turn out to be more robust in facilitating L2 vocabulary learning than subtitles (L1). Although a greater number of studies claimed that types of on-screen texts had little to do with the learning outcome, some defects in these studies cannot be ignored, such as the appropriateness of the difficulty of audiovisual input and the feasibility of the test procedure. What is more, there is a paucity of data about the newly emerging dual subtitles. So it is too early to draw a conclusion on the optimum type of on-screen text. Second, concerning the various modes of captions, it is more difficult to announce which one is superior to the others in aiding L2 vocabulary development. Nevertheless, some indications may be drawn from the present studies: (1) the dispute over the superiority of captioning integrity or visual salience *via* reduction has not been solved; (2) glosses and annotations which provide opportune and accessible meanings of the target words are conducive to the construction of form-meaning connection; (3) enhancement which has yielded mixed results—effective in the short term but ineffective in the long term—may be accompanied with other techniques, such as glosses and annotations to increase the overall effectiveness. Third, apart from the foci of this review (i.e., types of on-screen texts and modes of captions), some learner-related factors (e.g., vocabulary size and L2 proficiency) share similar influence on L2 word learning in audiovisual input with those in written input. And due to the nature of audiovisual input, there are quite a number of experiment-related factors to be explored, among which some factors related to learning materials, instructional strategies, and test time have already been taken into consideration, while more are underexplored and call for more specific and accurate experimental designs.

Since the initial shift of subtitles and captions to the SLA and the CALL domains, researchers have devoted themselves to investigating the functions of such on-screen texts in almost every aspect of L2 learning, especially vocabulary development. They first studied subtitles and captions separately and reported their benefits in enhancing vocabulary learning outcomes, which provided insights for the following in-depth research. Consequently, the past 10 years witnessed a tendency that a growing number of studies have endeavored to find the most beneficial type of on-screen text, namely, the most suitable language to be displayed on the screen. When the role of captions was gradually confirmed, researchers have turned to explore the rewarding way of displaying these captions (i.e., modes of captions).

This tendency implies that on-screen texts are of great potential in facilitating L2 vocabulary learning on the whole. The problem is how to fully tap into their potentials (i.e., in proper language, with suitable displaying mode, and to various groups of learners). Hence, researchers are encouraged to look more deeply into this field in the future and suggestions are provided in the following respects. First of all, as for the proper language, even though subtitles and captions have been extensively examined, dual subtitles, the newly emerging type of on-screen text which may combine the boon of both subtitles and captions, need to be further explored. Secondly, in terms of the suitable displaying mode of captions, the superiority of integrity or visual salience *via* reduction is still open to debate. We will wait and see more studies to be engaged and expect clear-cut recommendations for L2 teaching and learning. Thirdly, regarding the various groups of learners, the bulk of the current studies were conducted in an EFL context which invariably took English as their target language, only with a few exceptions (e.g., Sydorenko, 2010; Bisson et al., 2014; Montero Perez et al., 2014b, 2015, 2018; Cintrón-Valentín and Garciá-Amaya, 2021). The situation may be counterbalanced by introducing various second languages to English speakers (e.g., Winke et al., 2010, 2013). Moreover, the majority of the native languages belonged to the same language family—the Indo-European family, for example, Dutch, French, Spanish, and Norwegian. However, the distance between native and target languages may also influence the learning outcomes. Therefore, languages in different families (e.g., Chinese, Arabic, Finnish, and Japanese) also deserve attention. Fourthly, as to the design of the research, some high-tech devices, such as eye-tracking technique, may supplement the off-line statistics derived from the traditional test format with online performance of the learners’ vocabulary process. Besides, most studies adopted a one–off intervention to tap into the role of audiovisual input in L2 vocabulary building, but it is also urgent to evaluate its long-term effect in that vocabulary learning “is an incremental process in which words should be encountered and retrieved repeatedly before they can be firmly entrenched in the mental lexicon” (Peters et al., 2016, p. 146). Fifthly, it is suggested that more studies be conducted to explore the effects of on-screen texts on the learning of formulaic sequences which comprise idioms, collocations, and other multiword units, as they are pervasive in authentic input and contribute to idiomatic L2 competence (Gholami, 2021a,b, 2022). Finally, new technologies dealing with different audiovisual input and their effectiveness need to be examined and reported timely so as to contribute to this promising area (e.g., Lin, 2021; Wu et al., 2021). Teaching with audiovisual input may turn out to be the next revolution in L2 vocabulary learning, since the ever-developing multimedia technology offers easy accessibility and simplified manipulation to teachers and learners to meet their requirements whenever and wherever possible.

## Author Contributions

RW contributed to the conceptualization, investigation, and writing—original draft. LF contributed to the conceptualization and writing—review and editing, and supervision. Both authors contributed to the article and approved the submitted version.

## Funding

This study was funded by Ma’anshan University under Project for Cultivating Outstanding Talents in Universities.

## Conflict of Interest

The authors declare that the research was conducted in the absence of any commercial or financial relationships that could be construed as a potential conflict of interest.

## Publisher’s Note

All claims expressed in this article are solely those of the authors and do not necessarily represent those of their affiliated organizations, or those of the publisher, the editors and the reviewers. Any product that may be evaluated in this article, or claim that may be made by its manufacturer, is not guaranteed or endorsed by the publisher.

## Footnotes

^1^“Audiovisual input” and “videos with on-screen texts” are used interchangeably in this review.

^2^Since this review focuses on vocabulary learning in L2 context, reversed subtitle which accompanies L1 video is ignored.

^3^“Vocabulary learning” and “vocabulary acquisition” are used interchangeably in this review, though minor difference exists between them, that is, the former usually stresses an intentional learning behavior while the latter refers to an incidental by-product of learning something else.

^4^Since on-screen text is the focus of this review, some empirical studies that investigated factors influencing L2 vocabulary learning by employing audiovisual input without any on-screen text will not be considered.
